# Characterization and Clonal Diffusion of Ceftaroline Non-Susceptible MRSA in Two Hospitals in Central Italy

**DOI:** 10.3390/antibiotics10081026

**Published:** 2021-08-23

**Authors:** Gianluca Morroni, Simona Fioriti, Federica Salari, Andrea Brenciani, Lucia Brescini, Marina Mingoia, Eleonora Giovanetti, Antonella Pocognoli, Andrea Giacometti, Elisa Molinelli, Annamaria Offidani, Oriana Simonetti, Oscar Cirioni

**Affiliations:** 1 Department of Biomedical Sciences and Public Health, Polytechnic University of Marche, 60126 Ancona, Italy; g.morroni@staff.univpm.it (G.M.); s.fioriti@staff.univpm.it (S.F.); federica.salari.2@gmail.com (F.S.); a.brenciani@staff.univpm.it (A.B.); l.brescini@staff.univpm.it (L.B.); m.mingoia@staff.univpm.it (M.M.); a.giacometti@staff.univpm.it (A.G.); o.cirioni@staff.univpm.it (O.C.); 2 Department of Life and Environmental Sciences, Polytechnic University of Marche, 60126 Ancona, Italy; e.giovanetti@staff.univpm.it; 3 Clinical Microbiology Laboratory, “Ospedali Riuniti”, 60126 Ancona, Italy; Antonella.Pocognoli@ospedaliriuniti.marche.it; 4 Dermatological Clinic, Department of Clinical and Molecular Sciences, Polytechnic University of Marche, 60126 Ancona, Italy; molinelli.elisa@gmail.com (E.M.); a.offidani@staff.univpm.it (A.O.)

**Keywords:** ceftaroline, MRSA, penicillin-binding proteins, SCC*mec*

## Abstract

Background: Ceftaroline represents a novel fifth-generation cephalosporin to treat infections caused by methicillin-resistant Staphylococcus aureus (MRSA). Methods: Ceftaroline susceptibility of 239 MRSA isolates was assessed by disk diffusion and a MIC test strip following both EUCAST and CLSI guidelines. Non-susceptible isolates were epidemiologically characterized by pulsed-field gel electrophoresis, spa typing, and multilocus sequence typing, and further investigated by PCR and whole genome sequencing to detect penicillin-binding protein (PBP) mutations as well as antibiotic resistance and virulence genes. Results: Fourteen isolates out of two hundred and thirty-nine (5.8%) were non-susceptible to ceftaroline (MIC > 1 mg/L), with differences between the EUCAST and CLSI interpretations. The characterized isolates belonged to seven different pulsotypes and three different clones (ST228/CC5-t041-SCC*mec*I, ST22/CC22-t18014-SCC*mec*IV, and ST22/CC22-t022-SCC*mec*IV), confirming a clonal diffusion of ceftaroline non-susceptible strains. Mutations in PBPs involved PBP2a for ST228-t041-SCC*mec*I strains and all the other PBPs for ST22-t18014-SCC*mec*IV and ST22-t022-SCC*mec*IV clones. All isolates harbored antibiotic resistance and virulence genes with a clonal distribution. Conclusion: Our study demonstrated that ceftaroline non-susceptibile isolates belonged not only to ST228 strains (the most widespread clone in Italy) but also to ST22, confirming the increasing role of these clones in hospital infections.

## 1. Introduction

*Staphylococcus aureus* is a common human commensal, but also one of the leading causes of human infections such as endocarditis, bacteremia, osteomyelitis, and skin as well as soft tissue infections [1]. The acquisition of the staphylococcal cassette chromosome *mec* (SCC*mec*) and the *mecA* gene confers resistance to β-lactams and prompts the diffusion of methicillin-resistant *Staphylococcus aureus* (MRSA) worldwide [2]. The success of MRSA as a pathogen is confirmed by the multiple epidemic waves that in the last years involved community settings as well [3]. Indeed, MRSA is a challenging pathogen, able to acquire different antibiotic resistance determinants and virulence factors [1]. Although some antibiotics used against MRSA, such as daptomycin or dalbavancin, are highly effective against staphylococcal infections [4,5,6,7], β-lactams remain clinically useful due to their mild side effects [8]. Development of new antibiotics against MRSA leads to the approval of two new cephalosporins, ceftobiprole and ceftaroline [9]. In particular, ceftaroline demonstrates a broad spectrum against both Gram-positive and Gram-negative bacteria [10]. This antibiotic is administered as a prodrug, ceftaroline fosamil, that is rapidly converted into its active form in the plasma [10]. Differently from the other β-lactams, ceftaroline binds penicillin-binding protein (PBP) 2a and PBP2x, and therefore is effective against MRSA and β-lactam-resistant *Streptococcus pneumoniae* [8]. Surveillance studies confirmed the efficacy of ceftaroline against MRSA and other Gram positives, but at the same time identified some non-susceptible and resistant strains [11,12,13]. Resistance mechanisms involve amino acid substitutions mainly in PBP2a, both in the non-penicillin-binding domain and in the transpeptidase domain [14,15]. Moreover, it has been demonstrated that substitutions in other PBPs seemed to play a role in ceftaroline resistance [16]. In Italy, studies on ceftaroline-resistant MRSA isolates are absent, apart the recent work from Bongiorno et al. that characterized a collection of Italian MRSA strains isolated in 2012 [17]. To gain more information about ceftaroline resistance in Italy, we performed surveillance on the activity of this antibiotic against clinical MRSA strains collected from two hospitals (the “Ospedali Riuniti” of Ancona and the “Engles Profili” of Fabriano) in Central Italy. Isolates that were determined to be non-susceptible to ceftaroline (MIC > 1 mg/L) were further investigated to disclose clonal relationships, identify mutations involved in ceftaroline resistance, and characterize antibiotic resistance and virulence traits.

## 2. Results and Discussion

### 2.1. Susceptibility to Ceftaroline

Disk diffusion assay results were different according to the method used (Table 1). Following the EUCAST guidelines, 41 isolates were classified as susceptible, increased exposure (I, 17.1%), and twelve strains were resistant (5.0%). Alternatively, following the CLSI guidelines, ten (4.1%) and one (0.4%) isolates were determined to be susceptible-dose dependent and resistant, respectively. All non-susceptible isolates identified with both disk diffusion methods were subjected to minimum inhibitory concentration (MIC) determination. Fourteen strains showed MIC > 1 mg/L (Table 1 and Table S1) and thus were classified as non-susceptible (5.8%). Seven isolates (2.9%) showed a MIC = 2 mg/L while the other seven strains (2.9%) had a MIC = 4 mg/L. MIC interpretation varied accordingly to EUCAST or CLSI guidelines. While EUCAST (for infections other than pneumonia) identified seven strains as resistant (MIC > 2 mg/L) and seven strains as susceptible, increased exposure (MIC > 1 mg/L), CLSI interpretation classified all strains as susceptible-dose dependent. These results highlighted different interpretations of the two institutes. Results from both CLSI methods were comparable, as already showed by Sader et al. [18], while EUCAST demonstrated higher differences between disk diffusion and MIC interpretations. Our data confirmed discrepancies in EUCAST disk diffusion assays compared to MIC determination (both with broth microdilution or a MIC test strip) as reported before [19], and further suggested the need of a revision of disk diffusion criteria. Overall, ceftaroline resistance rates in our collection were very low and comparable to other surveillance studies on MRSA and ceftaroline [13,20,21]. This trend derived also from the recent breakpoint changes made in 2019 [21,22] that established higher MIC breakpoints for ceftaroline resistance interpretation.

### 2.2. Epidemiology of Ceftaroline Non-Susceptible MRSA

**Figure 1:**
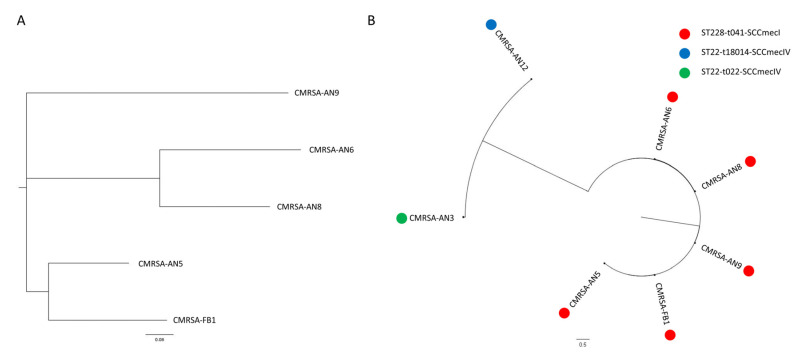
Phylogenetic tree of ceftaroline non-susceptible MRSA. Panel (**A**) shows the dendrogram, including only ST228-t041-SCC*mec*I, while panel (**B**) shows all isolates sequenced in the study. *Staphylococcus aureus* Mu50 was used as the reference genome.

The 14 isolates that showed ceftaroline MICs > 1 mg/L were further investigated. SmaI PFGE experiments detected seven different pulsotypes (A–G, Table S1). One strain for each pulsotype was subjected to whole genome sequencing (WGS). Five strains (showing pulsotypes A, B, D, E, and F) belonged to the same spa type (t041) and sequence type (ST228 and clonal complex CC5). The other two isolates (pulsotype C and G, respectively) shared the same ST (ST22, CC22) but different spa types, t18014 and t022, respectively (Table S1). ST228 isolates showed a SCC*mec* type I while ST22 strains harbored a SCC*mec* type IV (Table 2). Phylogenetic analysis confirmed that ST228-t041-SCC*mec*I isolates constituted a unique cluster, despite some variability (Figure 1A). Indeed, PFGE, a phylogenetic tree, and SNPs counts confirmed this diversity (SNPs min 327, max 624, mean 506, and median 475). Interestingly, strains collected from different hospitals showed the same PFGE pulsotype (i.e., pulsotype B), suggesting an inter-hospital transmission of these clones. Spread of identical clones in different hospitals has already been described in a previous work where the same linezolid-resistant *Staphylococcus epidermidis* clone was recovered in two hospitals of the same region [23]. The ST22 isolates constituted a different branch of the phylogenetic tree and demonstrated a higher diversity compared to the ST228-t041-SCC*mec*I isolates (SNPs 1192, Figure 1B), as also confirmed by the different spa type. Both STs represented clinical MRSA strains previously associated with ceftaroline resistance [24,25], although the recent paper from Bongiorno et al. [17] showed that resistant strains recovered in Italy belonged only to ST228. Our findings revealed that ceftaroline-resistant isolates also belong to ST22 and confirmed the ongoing increase of ST22 prevalence in Italian MRSA [26]. In our study, ceftaroline non-susceptible clones belonged to ST228 and ST22, while our previous work showed that ceftobiprole-resistant strains in one of the two hospitals (the “Ospedali Riuniti” of Ancona) also belonged to other STs (ST5, ST8, and ST4873) and harbored distinct PBP mutations [27]. Even though ceftaroline and ceftobiprole have similar mechanisms of action, these findings could suggest that resistance mechanisms varied within the two drugs for mutations and clones involved.

### 2.3. Analysis of PBP and GDPP Mutations

All the isolates harbored substitutions in the PBPs that might correlate with the non-susceptible phenotype (Table 2). The N146K mutation in PBP2a was found in all ST228-t041-SCC*mec*I isolates and has been previously associated with resistance to the new anti-staphylococcal cephalosporins ceftaroline [15] and ceftobiprole [25]. N146K substitution is localized in the allosteric non-penicillin-binding domain of PBP2a and, as other mutations in this region, could probably contribute to ceftaroline resistance [15]. Regarding the other PBPs and GDPP, the mutations differed among the ST228 isolates (Table 2). Interestingly, in CMRSA-AN9 and CMRSA-FB1 the only detected mutation was N146K in PBP2a. On the other hand, CMRSA-AN8 showed a stop mutation in GDPP, leading to a loss of protein function that correlates with an increased β-lactams resistance in MRSA [28,29]. The ST22 strain mutations were different. CMRSA-AN3 harbored the S225R substitution in PBP2a, a known mutation found in ceftobiprole-resistant MRSA [27], while CMRSA-AN12 showed a wild-type PBP2a. Furthermore, ST22 clones differed from the ST228 isolates in the number of mutations in the canonical PBPs (Table 2), which could correlate with the ceftaroline non-susceptible phenotype. The influence of amino acid substitutions in PBPs other than PBP2a could be particularly important in CMRSA-AN12 that harbored a wild-type PBP2a. Indeed, ceftaroline demonstrated affinity not only for PBP2a but also for PBP1, PBP2, and PBP3 [8]. Some of those mutations occurred in the transpeptidase domain of PBPs and have been previously detected in clinical MRSA strains with reduced susceptibility to ceftaroline belonging to the same ST [25]. In addition, the two ST22 clones showed the same ceftaroline MIC, although one of them harbored the S225R mutation in PBP2a (this substitution occurred in the non-penicillin-binding domain as N146K). It is reasonable to assume that this substitution has no influence in ceftaroline resistance, as previously reported in African MRSA susceptible to ceftaroline and ceftobiprole showing the S225R mutation [30].

### 2.4. Resistome and Virulence Analysis

Beside ceftaroline non-susceptibility, all characterized strains had additional resistance genes (Table S2). Genes conferring macrolide, lincosamide, and streptogramin B (MLS_B_) resistance, *erm*(A) and *erm*(C), were common in all isolates, while the aminoglycoside resistance determinants were found only in ST228-t041-SCC*mec*I isolates. These associations between resistance genes and certain antibiotics as well as clonal lineages have already been reported in the Italian survey of MRSA from pneumonia [26]. Notably, three T228-t041-SCC*mec*I isolates (i.e., CMRSA-AN6, CMRSA-AN8, and CMRSA-AN9) possessed the multidrug efflux pump gene *qacA* (CMRSA-AN6 also harbored the *qacG* gene) involved in resistance to organic cations, both monovalent and divalent [31]. These genes could contribute to the persistence of the strains, allowing the survival of these clones in hospital settings. Moreover, CMRSA-AN6 represented the isolate with the highest number of virulence factors and the only one expressing the leucotoxin *lukE*-*lukD*, a bi-component leucotoxin that induced dermonecrosis but showed lower toxicity compared to other staphylococcal leucotoxins [32]. Enterotoxins were expressed by all isolates following the same clonal distribution already described by Antonelli et al. [26]. ST228 carried *sea* and *seo* genes (CMRSA-AN6 also carried *seg*, *sei*, * sem*, *sen*, and *seu* genes) while ST22 harbored *seo*, *sec*, *seg*, *sei*, *sem*, *sen*, and *seu* genes. Our results further confirmed a relationship between virulence genes and clones, suggesting a stronger adaptive capability of ST22 to clinical settings compared to ST228 [31]. CMRSA-AN6 constituted an exception. Even though it belonged to ST228, it possessed the highest number of virulence genes and demonstrated that clones considered less virulent can also acquire virulence traits.

Ceftaroline is a novel cephalosporin, highly effective against MRSA and with a low prevalence of resistance. Resistance mechanisms included mutations in all PBPs and involved hospital clones of MRSA, such as CC5, CC8, or CC22. Our study depicted the situation in two hospitals of Central Italy, confirming a low resistance rate to ceftaroline in clinical MRSA. Non-susceptible isolates were rare and belonged to hospital clones already known and diffused worldwide. Conversely from a previous Italian report [17], low-resistance MRSA strains in our settings also involved ST22/CC22 clones (never associated with low ceftaroline resistance in our country), which in the last few years replaced the ST228 clone in Italy [26]. Although our findings represent the situation in a small region, they suggest that surveillance studies on the rise of ST22 and its association with ceftaroline resistance should be performed, also considering the higher adaptive capability of the ST22 clone [33]. Monitoring resistance and its mechanisms is pivotal to avoid the diffusion of resistance clones and preserve the activity of this cephalosporin.

## 3. Materials and Methods

### 3.1. Strain

From March 2018 to December 2019, 239 MRSA isolates were collected at the Clinical Microbiology Laboratory of the “Ospedali Riuniti” hospital, Ancona (AN), and at the “Engles Profili” hospital, Fabriano (FB). All isolates were included in the study regardless of the clinical sample. The bacterial isolates were identified by MALDI-TOF; resistance to methicillin was confirmed by the cefoxitin disk test and GeneXpert on the *mecA* gene. To prevent duplicate isolates, only one strain for each patient was included in this study.

### 3.2. Susceptibility to Ceftaroline and MIC Determination

MRSAs were tested for ceftaroline resistance by disk diffusion assays and interpreted following both CLSI and EUCAST guidelines (Table 1) [34,35]. The isolates determined to be non-susceptible or resistant by disk diffusion were further subjected to MIC determination using a MIC test strip (Liofilchem, Roseto degli Abruzzi, Italy), with three different experiments. *S. aureus* ATCC 29,213 and *S. aureus* ATCC 25,923 were used as quality controls.

### 3.3. Typing, WGS, and Genome Analysis

Non-susceptible strains were typed by SmaI PFGE [36]. One isolate for each pulsotype was subjected to WGS and subsequent analysis. DNA sequencing was performed using the long-reads approach of Nanopore MiniON (Oxford Nanopore). Raw reads were assembled using canu software v.2.1.1 [37]. Genomes were used to draw a phylogenetic tree and calculate SNPs using CSIPhylogeny v.1.4 (https://cge.cbs.dtu.dk/services/CSIPhylogeny/ (accessed on 15 April 2021)), and to type strains through spa typing [27], MLST [38], and SCC*mec* cassettes via SCC*mec* finder v.1.2 (https://cge.cbs.dtu.dk/services/SCCmecFinder/ (accessed on 15 April 2021)). Moreover, antibiotic resistance genes were found using Resfinder 4.1 (https://cge.cbs.dtu.dk/services/ResFinder/ (accessed on 15 April 2021)) and virulence determinants were studied by VirulenceFinder 2.0 (https://cge.cbs.dtu.dk/services/VirulenceFinder/ (accessed on 15 April 2021)).

### 3.4. Analysis of PBP Mutations

Amplification of *mec*A, *pbp*1, *pbp*2, *pbp*3, and *pbp*4 genes was performed using primers previously described [27], while the *gdpP* gene was amplified with gdpP-FW (CATCTATCGTTCTTGTCGT) and gdpP-RV (ATTGTGCTATCGCCTCTTC). Amplicons were sequenced by the Sanger approach and compared with the reference genome of the methicillin-resistant and ceftaroline-susceptible *S. aureus* Mu50 (acc. No. BA000017.4).

## Figures and Tables

**Table 1:** Results of disk diffusion assays and MIC determination following EUCAST or CLSI methods and interpretations.

Isolate Category	Breakpoint Diameters (mm)		No. of Isolates Tested (% on Total Isolates of the Study)			
	EUCAST	CLSI	DD ^a^ (EUCAST)	DD ^a^ (CLSI)	MIC ^b^ (EUCAST)	MIC ^b^ (CLSI)
Susceptible	≥20	≥25	186 (77.8%)	228 (95.4%)	39 (94.1%)	39 (94.1%)
Susceptible, increased exposure	17–19	NA	41 (17.2%)	NA	7 (2.9%)	NA
Susceptible-dose dependent	NA	20–24	NA	10 (4.2%)	NA	14 (5.8%)
Resistant	<17	≤19	12 (5.0%)	1 (0.4%)	7 (2.9%)	0 (0%)

^a^, Disk diffusion. ^b^, MIC determinations were performed on 53 isolates determined to be non-susceptible after disk diffusion screening. NA, not applicable.

**Table 2:** Genetic characterization of ceftaroline non-susceptible MRSA isolates.

Strain	MIC (mg/L)	Amino Acid Substitutions						PFGE Pulsotype	Spa Type	MLST	SCC*mec*
		PBP2a	PBP1	PBP2	PBP3	PBP4	GDPP				
CMRSA-AN3	2	S225R	S629-S664T	C197Y-L246V-P285A-T439V-T691A	R504K-K584N	D98E-S189T-E398A	I52V-N105D-S391P	C	t18014	ST22	IV
CMRSA-AN5	2	N146K	WT	C197Y	T92X	WT	WT	A	t041	ST228	I
CMRSA-AN6	4	N146K	S194N	C197Y	WT	N337D	WT	D	t041	ST228	I
CMRSA-AN8	2	N146K	WT	C197Y	WT	L354X	Truncated	E	t041	ST228	I
CMRSA-AN9	4	N146K	WT	WT	WT	WT	WT	F	t041	ST228	I
CMRSA-AN12	2	WT	S629T-S664T	C197Y-L246T-P285A-T439V-T691A	R504K-K584N	D98E-S189T-E398A	I52V-N105D-S391P	G	t022	ST22	IV
CMRSA-FB1	2	N146K	WT	WT	WT	WT	WT	B	t041	ST228	I

## Data Availability

The data presented in this study are available on request from the corresponding author.

## Supplementary Materials

The following are available online at https://www.mdpi.com/article/10.3390/antibiotics10081026/s1. Table S1. Epidemiological characteristics of ceftaroline non-susceptible isolates. Table S2. Antibiotic resistance genes and virulence factors of ceftaroline non-susceptible MRSA.
