# Supplementary material for: Characterization and Clonal Diffusion of Ceftaroline Non-Susceptible MRSA in Two Hospitals in Central Italy
Source: Antibiotics (Basel). 2021 Aug 23;10(8):1026. doi: 10.3390/antibiotics10081026 (PMC8388857; doi:10.3390/antibiotics10081026)
Supplement: Supplementary file 1 [file antibiotics-10-01026-s001.zip › antibiotics-1340493-supplementary.pdf]

Table S1. Epidemiological characteristics of ceftaroline non-susceptible isolates.

**Strains**	**Origin**	**MIC (mg/L)**	**PFGE pulsotype**	**Spa-typing**	**MLST (CC)**
CMRSA-AN1	Ancona	2	A	t041	ST228 (CC5)
CMRSA-AN2	Ancona	4	B	t041	ST228 (CC5)
CMRSA-AN3	Ancona	2	C	t18014	ST22 (CC22)
CMRSA-AN4	Ancona	4	A	t041	ST228 (CC5)
CMRSA-AN5	Ancona	2	A	t041	ST228 (CC5)
CMRSA-AN6	Ancona	4	D	t041	ST228 (CC5)
CMRSA-AN7	Ancona	4	D	t041	ST228 (CC5)
CMRSA-AN8	Ancona	2	E	t041	ST228 (CC5)
CMRSA-AN9	Ancona	4	F	t041	ST228 (CC5)
CMRSA-AN10	Ancona	2	B	t041	ST228 (CC5)
CMRSA-AN11	Ancona	4	F	t041	ST228 (CC5)
CMRSA-AN12	Ancona	2	G	t022	ST22 (CC22)
CMRSA-FB1	Fabriano	2	B	t041	ST228 (CC5)
CMRSA-FB2	Fabriano	4	B	t041	ST228 (CC5)

Table S2. Antibiotics resistance genes and virulence factors of ceftaroline non-susceptible MRSA.

**Strain**	**Spa-type**	**MLST**	**Antibiotics resistance genes**				**Disinfectants resistance genes**	**Virulence factors**
			**β-lactams**	**MLS_B**	**Aminoglicoside**	**Chlorampenicol**		
**CMRSA-AN3**	t18014	ST22	*mecA, blaZ*	*erm*(C)	-	*cat*pC221	-	*sak, scn, aur, hlgA, hlgB, hlgC, seo, sec, seg, sei, sem, sen, seu*
**CMRSA-AN5**	t041	ST228	*mecA*	*erm*(A)	*aac*(6')-*aph*(2''), *ant*(6)-Ia, *aph*(3')-III, *ant*(9)-Ia	-	-	*sak, scn, aur, splA, splB, hlgA, hlgB, hlgC, sea, seo*
**CMRSA-AN6**	t041	ST228	*blaZ, mecA*	*erm*(A)	*aac*(6')-*aph*(2''), *ant*(6)-Ia, *aph*(3')-III, *ant*(9)-Ia	-	*qacA, qacG*	*sak, scn, aur, splA, splB, hlgA, hlgB, hlgC, sea, seo, sec, seg, sei, sem, sen, seu, lukD, LukE*
**CMRSA-AN8**	t041	ST228	*blaZ, mecA*	*erm*(A)	*aac*(6')-*aph*(2''), *ant*(6)-Ia, *aph*(3')-III, *ant*(9)-Ia	*cat*pC221	*qacA*	*sak, scn, aur, splA, splB, hlgA, hlgB, hlgC, sea, seo*
**CMRSA-AN9**	t041	ST228	*blaZ, mecA*	*erm*(A)	*aac*(6')-*aph*(2''), *ant*(6)-Ia, *aph*(3')-III, *ant*(9)-Ia	-	*qacA*	*sak, scn, aur, splA, splB, hlgA, hlgB, hlgC, sea, seo*
**CMRSA-AN12**	t022	ST22	*mecA, blaZ*	*erm*(C)	-	-	-	*sak, scn, aur, hlgA, hlgB, hlgC, seo, sec, seg, sei, sem, sen, seu*
**CMRSA-FB1**	t041	ST228	*mecA, blaZ*	*erm*(A)	*aac*(6')-*aph*(2''), *ant*(6)-Ia, *aph*(3')-III	-	-	*sak, scn, aur, splA, splB, hlgA, hlgB, hlgC, sea, seo*
